# Chitosan Nanoparticles Loaded with Alkaline-Released Rice Bran Hydroxycinnamic Acids: Physicochemical Characterisation, Antioxidant Activity and In Vitro Bioaccessibility

**DOI:** 10.3390/foods15203745

**Published:** 2026-10-21

**Authors:** Georgia Lazaridou, Nikolaos Nenadis

**Affiliations:** 1 Laboratory of Food Chemistry & Technology, School of Chemistry, Aristotle University of Thessaloniki, 54124 Thessaloniki, Greece; 2 Natural Products Research Centre of Excellence-AUTH (NatPro-AUTH), Center for Interdisciplinary Research and Innovation (CIRI-AUTH), 57001 Thessaloniki, Greece

**Keywords:** rice bran, ferulic acid, *p*-coumaric acid, ionic gelation, chitosan nanoparticles, bioaccessibility

## Abstract

Food-grade nanocarriers are widely investigated for improving the stability and bioaccessibility of labile phenolics. Ferulic and *p*-coumaric acid, the principal hydroxycinnamic acids of rice bran, were recovered from this rice milling by-product and encapsulated in chitosan nanoparticles by ionic gelation with sodium tripolyphosphate. Because they occur mainly esterified to cell-wall polysaccharides, aqueous ethanolic extraction, acid hydrolysis and alkaline hydrolysis were compared as release routes; alkaline hydrolysis gave the highest total phenolic content (4.58 mg gallic acid equivalents per g), cupric reducing capacity and by far the highest levels of both acids. This extract was encapsulated at three phenolic loadings; the 1.5 mg GAE/mL loading was the highest loading that remained nanoscale with polydispersity below 0.4. The resulting particles had a mean diameter of 184.7 nm, a polydispersity index of 0.35 and a zeta potential of +32.8 mV, retained on loading, and appeared under electron microscopy as discrete spheroidal particles of 100–200 nm that were absent from the blanks. Encapsulation efficiency was highest for this formulation, 34.6% by Folin–Ciocalteu, and 42.6% and 36.4% for *p*-coumaric and ferulic acid by UHPLC-DAD. A synthetic mixture matched to the extract was encapsulated more efficiently (52.3% and 45.3%, respectively), supporting an inhibitory effect of the extract matrix. FTIR, XRD and TGA were consistent with entrapment, reduced crystallinity and altered thermal decomposition relative to chitosan. Per unit of phenolic content, the reducing capacity of the nanoparticles matched that of the free extract, so that encapsulation preserved intrinsic antioxidant activity. Under simulated digestion, intestinal phase recovery of both acids was approximately 4-fold that ofthe free extract (*p* < 0.001), with antioxidant recovery likewise being higher. Chitosan nanoparticles are therefore promising for valorising rice bran phenolics in functional foods and nutraceuticals.

## 1. Introduction

Rice (*Oryza sativa* L.) is among the most widely cultivated cereals worldwide and a staple food for more than half of the global population. Global paddy production reached approximately 805 million tonnes in 2023, close to 90% of it in Asia [1]. Milling generates several by-product streams, principally hulls and bran, which are conventionally handled as low-value agricultural residues despite containing appreciable quantities of bioactive constituents. Their recovery aligns with the European Green Deal and specifically with the Circular Economy Action Plan [2] and the Farm to Fork Strategy [3], which identify the valorisation of agri-food side streams as a priority [3].

Rice bran, one of the principal rice milling by-products, accounts for approximately 8–10% of the weight of rough rice [4], corresponding to an estimated 64–80 million tonnes generated annually. It is a particularly rich source of hydroxycinnamic acids, mainly ferulic and *p*-coumaric acid, present at approximately 68.5–179.3 and 13.1–63.5 mg/100 g dry weight, respectively [5]. Both act as antioxidants whose activity is governed by the substitution pattern of the aromatic ring [6], and both have attracted interest as candidates for protective delivery systems: ferulic acid in connection with neuroprotective and antidiabetic effects [7,8] and *p*-coumaric acid in relation to the improvement of its physicochemical behaviour [9]. Critically, these acids occur mainly esterified to arabinoxylans and other cell-wall polysaccharides, from which they must be liberated before they can be efficiently recovered [10].

Globally, rice bran remains underutilised, largely as animal feed; the main exception is oil extraction in the major Asian producing countries, where even the defatted residue is treated as low-value waste rather than a source of phenolics [11]. The same pattern holds in European producing countries. In Greece, for example, an annual paddy production of approximately 222,000 tonnes [12] corresponds to a bran stream of roughly 20,000 tonnes, destined largely for animal feed. Wherever rice is milled, therefore, the bran constitutes a compositionally well-characterised feedstock for which higher-value applications remain limited.

Several factors constrain the practical use of rice bran phenolics once recovered, including susceptibility to oxidative and photolytic degradation under typical processing and storage conditions [13], poor aqueous solubility [9] and limited bioaccessibility during gastrointestinal passage. Encapsulation at the micro- and nanoscale has been widely investigated to address these constraints, with nanoscale systems attracting particular attention for their high surface-to-volume ratio and reported capacity to protect labile compounds during processing and gastrointestinal passage [14]. What has not been explored, however, is whether other constituents of the rice bran matrix interfere with particle formation. Such constituents are co-liberated or generated by the non-selective treatments used to release the bound hydroxycinnamic acids when a high phenolic yield is the objective.

Research on the nanoencapsulation of rice bran bioactives has concentrated almost exclusively on the lipophilic fraction, and in particular on γ-oryzanol and rice bran oil, for which lipid-based systems predominate. These include nanoemulsions stabilised by synthetic surfactants, by protein–surfactant combinations or by protein–polysaccharide mixtures [15,16,17,18], nanodispersions prepared by solvent displacement [19] and nanostructured lipid carriers affording controlled release in simulated intestinal fluid [20]. Individual constituents have been treated similarly, with encapsulation reported for ferulic acid [7] and for rice bran oil formulated for dermal protection against UV-B-induced injury [21]. Chitosan has appeared in this literature only in ancillary roles, as a reinforcing or coating agent on γ-oryzanol-loaded calcium pectinate microparticles [22,23] and as a shell on polymeric micelles carrying the same compound [24]. The polar phenolic fraction has received considerably less attention. Work to date has addressed the microencapsulation of soluble (free) phenolics and anthocyanins from white or pigmented varieties in gelatine, gum arabic or starch [25,26] and, at the nanoscale, a single surfactant-stabilised oil-in-water nanoemulsion of a black rice bran extract obtained by conventional aqueous ethanolic extraction and therefore comprising the free phenolic and anthocyanin fraction [27]. To the best of our knowledge, neither the use of chitosan as the particle-forming matrix for rice bran phenolics nor the nanoencapsulation of the bound hydroxycinnamic acid fraction liberated by alkaline hydrolysis has previously been reported. The present study addresses both gaps.

Chitosan, obtained by the deacetylation of chitin, is among the most extensively studied wall materials for encapsulation, owing to its biodegradability, biocompatibility, low toxicity and mucoadhesive character [14]. Unlike neutral carriers such as starch or maltodextrin, its protonated amino groups form networks spontaneously with polyanionic crosslinkers under mild aqueous conditions. In this way, the heat and organic solvents required by several alternative systems are avoided, an advantage when handling thermolabile phenolic acids. Ionic gelation with sodium tripolyphosphate (TPP) is the most widely used of these approaches, offering simplicity, low cost and the absence of toxic reagents [28]; the size, surface charge and network density of the resulting particles can be modulated through the polymer-to-crosslinker ratio and the pH of the medium [29].

For food applications, the origin of the chitosan is not a trivial consideration. Crustacean-derived chitosan falls within the scope of Annex II of Regulation (EU) No 1169/2011 [30] on the provision of food information to consumers and requires corresponding allergen labelling, whereas chitosan of fungal origin has been evaluated through the novel food procedure established by Regulation (EU) 2015/2283 [31]. Engineered nanomaterials intended for use in food are likewise covered by Regulation (EU) 2015/2283 [31] and are subject to the guidance of the European Food Safety Authority on nanoscience risk assessment in the food and feed chain [32]. The chitosan employed in the present work was crustacean-derived; these considerations are directly relevant to any subsequent translation of the system described here into commercial functional food products.

The present study therefore aimed to develop and characterise chitosan nanoparticles loaded with a polar rice bran extract enriched in ferulic and *p*-coumaric acids. Bound phenolics were released by alkaline hydrolysis following a previously optimised protocol [10], and encapsulation was performed by ionic gelation with TPP. The resulting nanoparticles were characterised with respect to particle size distribution, polydispersity, zeta potential and morphology; thermal stability (thermogravimetric analysis); crystalline structure (X-ray diffraction); and molecular interactions between extract and carrier (ATR-FTIR spectroscopy). Encapsulation efficiency was determined in parallel by a colorimetric (Folin–Ciocalteu) and a chromatographic (UHPLC-DAD) method, permitting direct comparison of the two approaches. In addition, a synthetic mixture of ferulic and *p*-coumaric acid standards, formulated to match the composition of the extract, was encapsulated under identical conditions as a matrix control to distinguish the behaviour of the target phenolic acids from that of the co-extracted constituents of the alkaline hydrolysate. Antioxidant capacity was assessed using in vitro assays (ABTS•^+^, DPPH• and CUPRAC), and simulated gastrointestinal digestion was applied to evaluate the effect of nanoencapsulation on phenolic bioaccessibility relative to the free extract. The findings are intended to support the valorisation of rice bran as a source of bioactive phenolic acids for functional food and nutraceutical applications.

## 2. Materials and Methods

### 2.1. Materials

This study did not involve human participants or animals, and no ethical approval was required.

#### 2.1.1. Raw Material

Rice bran (*Oryza sativa* L.), a mixture of the local cultivars (Nychaki and Glasé, as designated by the producer), was kindly provided by Begas Agro S.A. (Chalastra, Thessaloniki, Greece). The material was collected in November 2023, had not been subjected to heat stabilisation prior to delivery and was used as received, without defatting. It was transported under refrigeration, immediately stored at −20 °C in sealed glass containers without headspace and kept frozen until processing. Prior to extraction, the bran was sieved to a particle size below 500 µm. Moisture content was determined in triplicate using a DAB 100-3 near-infrared moisture analyser (Kern & Sohn GmbH, Balingen, Germany) operating at 105 °C to constant weight, and it was 12.0 ± 0.3% (*w*/*w*). Unless otherwise stated, all results are expressed per gram of rice bran as received.

#### 2.1.2. Chemicals and Reagents

ABTS (2,2′-azinobis(3-ethylbenzothiazoline-6-sulphonic acid) diammonium salt), α-amylase (from porcine pancreas; ≥5 U/mg), ferulic acid (≥98%), gallic acid, pancreatin (from porcine pancreas; 8× USP specification), *p*-coumaric acid (≥98%), pepsin (from porcine gastric mucosa; ≥250 U/mg), porcine bile extract, phosphoric acid, sulphuric acid and Trolox (6-hydroxy-2,5,7,8-tetramethylchroman-2-carboxylic acid) were purchased from Sigma-Aldrich (St. Louis, MO, USA). Ammonium acetate, copper (II) chloride dihydrate, disodium hydrogen phosphate, ethanol, ethyl acetate, Folin–Ciocalteu phenol reagent, HPLC-grade methanol, hydrochloric acid, monopotassium phosphate, neocuproine, potassium chloride, sodium carbonate, sodium chloride, anhydrous sodium sulphate, and HPLC-grade water (<1 µS/cm) were supplied by Chem-Lab (Zedelgem, Belgium). Potassium persulphate, sodium bicarbonate and sodium hydroxide were obtained from Merck (Darmstadt, Germany). DPPH (1,1-diphenyl-2-picrylhydrazyl) was purchased from TCI Chemicals (Tokyo, Japan). Chitosan (crustacean-derived; viscosity-average molecular weight 190–310 kDa; degree of deacetylation >90%; food grade) was supplied by Siveele B.V. (Breda, The Netherlands) and acetic acid by Carlo-Erba (Emmendingen, Germany). All other reagents were of analytical grade.

### 2.2. Preparation of Rice Bran Extracts

For the characterisation of the three extracts, three independent extractions were carried out and combined into a single pooled extract, which was subsequently analysed in triplicate.

#### 2.2.1. Extraction of Free Phenolics

Free phenolics were extracted according to Irakli et al. [10] with minor modifications. Powdered rice bran (1.00 g) was sonicated with 20 mL of 60% (*v*/*v*) aqueous ethanol for 10 min in an ultrasonic bath (Elmasonic S 60 H, Elma Schmidbauer GmbH, Singen, Germany; 37 kHz, 150 W) at room temperature. The mixture was centrifuged at 3260× *g* for 5 min at 4 °C (Thermo Scientific SL 16R, Thermo Fisher Scientific, Waltham, MA, USA), and the supernatant was filtered through a 0.45 µm hydrophilic PTFE membrane (Target Analysis, Thessaloniki, Greece). Extracts were stored at 4 °C in amber vials and analysed within 24 h.

#### 2.2.2. Recovery of Bound Phenolics by Alkaline Hydrolysis

Bound phenolics were released following the alkaline hydrolysis protocol of Irakli et al. [10] with slight modifications. Powdered rice bran (1.00 g) was treated with 25 mL of 4 M NaOH and sonicated for 1.5 h at 30 °C under a nitrogen atmosphere; the bath water was replaced every 30 min to maintain the temperature. The mixture was centrifuged (4696× *g*, 20 min, 4 °C) and the supernatant acidified to pH 2.0 with 12 M HCl in an ice bath. After a second centrifugation (4696× *g*, 20 min, 4 °C), the supernatant was extracted three times with 50 mL of ethyl acetate. The combined organic layers were dried over anhydrous sodium sulphate, decanted from the drying agent into a clean flask and evaporated to dryness under reduced pressure at 37 °C (Rotavapor R-200, Büchi Labortechnik AG, Flawil, Switzerland). The residue was reconstituted in 4 mL of methanol, filtered through a 0.45 µm PTFE membrane, and stored at −20 °C in amber vials until analysis. The dry residue amounted to 12.8% (*w*/*w*) of the rice bran. Contents are reported per gram of rice bran, the basis on which the three extraction routes are compared. This alkaline extract (AHE) was used for all subsequent encapsulation experiments; extracts from several such preparations were pooled and, where required, concentrated to obtain the phenolic loadings described in Section 2.4.

#### 2.2.3. Recovery of Bound Phenolics by Acidic Hydrolysis

For comparison, bound phenolics were also recovered by acid hydrolysis, adapted from Xu et al. [33], who optimised sulphuric acid extraction of bound phenolics from hulless barley bran; the sonication time was matched to that of the alkaline hydrolysis so that the two hydrolysates could be compared under an equivalent treatment duration. Powdered rice bran (1.00 g) was sonicated in 17 mL of 2 M H_2_SO_4_ for 1.5 h at 75 °C. The mixture was centrifuged (4696× *g*, 20 min, 4 °C) and the supernatant extracted three times with 50 mL of ethyl acetate. The combined organic phases were dried over anhydrous sodium sulphate, decanted from the drying agent into a clean flask and evaporated to dryness under reduced pressure at 37 °C. The residue was reconstituted in 4 mL of methanol, filtered (0.45 µm) and stored at −20 °C until analysis. The dry residue amounted to 5.9% (*w*/*w*) of the rice bran.

### 2.3. Analytical Procedures

#### 2.3.1. Total Phenolic Content

Total phenolic content (TPC) was determined by the Folin–Ciocalteu assay. In a 10 mL volumetric flask, 5 mL of water, 100 µL of aqueous ethanolic extract (free phenolics) or 25 µL methanolic extract (bound phenolics) and 0.5 mL of Folin–Ciocalteu reagent were combined. The two aliquot volumes differ because the hydrolysates are substantially richer in phenolics than the aqueous ethanolic extract; the smaller volume was required to keep the absorbance within the linear range of the calibration curve. After 3 min, 1.5 mL of 20% (*w*/*v*) Na_2_CO_3_ was added and the volume made up with water. Samples were incubated for 1 h in the dark at room temperature, and absorbance was measured at 750 nm (UV-1800 spectrophotometer, Shimadzu, Kyoto, Japan) against a reagent blank prepared with the corresponding solvent. Gallic acid standards were prepared in methanol and carried through the identical procedure; a total of 200 µL of solutions of 20–500 mg/L were taken, so that the response was linear over 4–100 µg gallic acid per 10 mL of reaction volume, that is 0.4–10 µg/mL in the final mixture (*y* = 0.0116*x* + 0.0145, R^2^ = 0.9998, with *x* in µg per 10 mL). Results are expressed as mg of gallic acid equivalents per g of rice bran (mg GAE/g). All determinations were performed in triplicate and results are reported as mean ± SD.

#### 2.3.2. Antioxidant Activity

Antioxidant activity was assessed by three complementary assays, with results expressed as µmol Trolox equivalents per g of rice bran (µmol TE/g). All determinations were performed in triplicate and results are reported as mean ± SD.

ABTS•^+^ scavenging activity. Assessed following Re et al. [34] with modifications. The radical cation was generated by reacting 7 mM ABTS with 140 mM potassium persulphate for at least 16 h in the dark, then diluted with phosphate-buffered saline (pH 7.4) to an absorbance of 0.70 ± 0.05 at 734 nm. Extract (20 µL of aqueous ethanolic, or 20 µL of a 1:10 methanolic dilution) was added to 2 mL of the diluted radical solution, and absorbance at 734 nm was recorded at 0 and 6 min. Inhibition was calculated as %Inh = [(*A*_0_ − *A*_t_)/*A*_0_] × 100, where *A*_0_ is the absorbance of the diluted radical solution at 0 min and *A*_t_ the absorbance recorded after 6 min of reaction with the sample. Trolox response was linear over 0.1–2.0 µmol per 2 mL of reaction volume (*y* = 1.96*x* − 2.1124, R^2^ = 0.9946).

DPPH• scavenging activity. Performed according to Nenadis and Tsimidou [35] with modifications. Extract (100 µL of aqueous ethanolic or 40 µL of methanolic) was mixed with 2.9 mL of 0.1 mM methanolic DPPH•, and absorbance at 516 nm was recorded at 0 and 30 min at room temperature. Scavenging activity was calculated as %RSA = [(*A*_0_ − *A*_t_)/*A*_0_] × 100, where *A*_0_ is the absorbance of the DPPH• solution at 0 min and *A*_t_ is the absorbance recorded after 30 min in the presence of the sample. Trolox response was linear over 0.1–1.2 µmol per 3 mL of reaction volume (*y* = 0.7861*x* + 0.3781, R^2^ = 0.9962).

CUPRAC assay. Measured following Apak et al. [36]. A mixture of 1 mL of 0.01 M CuCl_2_, 1 mL of 7.5 mM neocuproine, and 1 mL of 1 M ammonium acetate received 100 µL of aqueous ethanolic or 20 µL of methanolic extract, adjusted to 4.1 mL with water. After 30 min in the dark, absorbance was recorded at 450 nm against a reagent blank. Trolox response was linear over 1–8 µmol per 4.1 mL of reaction volume (*y* = 3.2430*x* + 0.0466, R^2^ = 0.9948).

In every assay, a reagent blank, prepared with the corresponding solvent in place of the extract, was read at the assay wavelength and subtracted.

#### 2.3.3. Determination of Phenolic Acids by UHPLC-DAD

Ferulic and *p*-coumaric acid contents were determined using a Shimadzu Nexera X2 UHPLC system (LC-30AD pump, SIL-20AC autosampler, CTO-20AC column oven, SPD-M30A photodiode array detector; Shimadzu Corporation, Kyoto, Japan). Separation was achieved on an Accucore C18 column (100 × 4.6 mm, 2.6 µm; Thermo Scientific, Waltham, MA, USA) at 35 °C, with isocratic elution using methanol/water containing 0.2% (*v*/*v*) phosphoric acid (24.5:75.5, *v*/*v*) at 0.5 mL/min. The injection volume was 1 µL for calibration standards and 5 µL for sample extracts, and the run time was 20 min. Detection was at 310 nm (*p*-coumaric acid) and 322 nm (ferulic acid). Compounds were identified by retention time and UV spectrum against authentic standards, and peak purity was confirmed from the photodiode array data. Calibration curves were constructed based on the mass injected rather than solution concentration. For *p*-coumaric acid (310 nm), *y* = 5399.4*x* − 710.68 (R^2^ = 0.9993) over 2.5–150 ng. For ferulic acid (322 nm), a curve fitted across the full standard range (2–300 ng) gave *y* = 10,361*x* − 112,116 (R^2^ = 0.9976); because the magnitude of its intercept limits accuracy at low response, a second curve restricted to 2–75 ng, *y* = 8107.5*x* − 6883.3 (R^2^ = 0.9999), was constructed and applied to all samples falling within that range, including the digestion series. The lowest calibration point of each curve was taken as the limit of quantification (2.5 and 2 ng for *p*-coumaric and ferulic acid, respectively). All samples were analysed in triplicate.

### 2.4. Preparation of Chitosan Nanoparticles

Chitosan nanoparticles (ChNPs) were produced by ionic gelation with TPP, following Stoica et al. [37] with modifications. Chitosan (1.75 mg/mL) was dissolved in 1% (*v*/*v*) acetic acid under magnetic stirring for 24 h at 25 °C, adjusted to pH 4.6 with 1 M NaOH, and filtered through a 0.45 µm membrane. A TPP solution (0.16 mg/mL in ultrapure water) was added dropwise at 1 mL/min under continuous stirring at 700 rpm, at a chitosan-to-TPP mass ratio of 3:1, for 30 min at 25 °C. For encapsulation, the phenolic extract was incorporated into the chitosan solution at a fixed volume fraction of 22% (*v*/*v*) prior to crosslinking. Three total phenolic loadings (1.0, 1.5 and 2.0 mg GAE/mL of final formulation) were obtained by adjusting the concentration of the extract rather than the volume added: extracts from several hydrolysis batches were pooled, concentrated under reduced pressure at 37 °C where a higher loading was required and diluted with methanol where a lower one was required. In every case, the extract and the standard mixture used for the matrix control were prepared in methanol (Section 2.2.2). Keeping the volume fraction constant ensured that the chitosan concentration, the chitosan-to-TPP ratio and the methanol content were identical across the three formulations. Because the loading was varied by concentrating or diluting the whole extract, the co-extracted constituents varied in proportion to the phenolic acids, so that the observed differences are attributable to the extract loading rather than to the phenolic concentration alone. The ionic strength of the medium was not measured. The blank nanoparticles were prepared with methanol in place of the extract at the same volume fraction, so that the methanol content was matched across all formulations. Once the optimal loading had been identified, a reference formulation containing *p*-coumaric and ferulic acid standards at concentrations matching those of the extract was prepared under identical conditions and used as a matrix control. In all preparations, the TPP solution was added to the chitosan solution; the reverse order of addition was not examined.

Each nanosuspension was divided into two fractions. The first was retained as a liquid suspension, without drying, for particle characterisation (size, polydispersity, and zeta potential; Section 2.5.1) and for the determination of encapsulation efficiency; an aliquot was centrifuged at 10,000× *g* for 50 min at 4 °C, and the supernatant was collected for quantification of non-encapsulated phenolics (Section 2.5.3). Centrifugation separated the nanoparticles from the continuous phase but did not yield enough solid material for the bulk solid-state analyses, and the second fraction was therefore freeze-dried directly (48 h; ALPHA 1-2 LDplus, Martin Christ, Osterode am Harz, Germany; −50 °C condenser, 0.1 mbar) to recover solid material for the remaining analyses. The resulting powder was hydrated in the minimum volume of ultrapure water required, without redissolution of the chitosan network; under these near-neutral conditions, the crosslinked particles remained intact, and their disruption for the release of the encapsulated phenolics required subsequent acidification (Section 2.5.6 and Section 2.6). This freeze-dried powder was used for solid-state characterisation (ATR-FTIR, XRD, TGA, SEM), the antioxidant assays of Section 2.5.6 and the digestion study (Section 2.6). The freeze-dried material was stored at −20 °C, protected from light, until analysis.

### 2.5. Characterisation of Nanoparticles

#### 2.5.1. Particle Size, Polydispersity and Zeta Potential

Hydrodynamic diameter, polydispersity index (PDI), and zeta potential were determined on the undiluted nanosuspensions by dynamic light scattering (Zetasizer Pro, Malvern Panalytical, Worcestershire, UK) at 25 °C, using a 632.8 nm laser and a detection angle of 173°. Samples were equilibrated for 120 s before measurement. Measurements were made without dilution since the backscatter detection geometry (173°) accommodates concentrated dispersions, and dilution would have altered the ionic strength and pH of the medium and hence the measured zeta potential. Material and dispersant refractive indices of 1.52 and 1.330 were applied, and zeta potential was measured in disposable folded capillary cells using the Smoluchowski approximation. All samples were analysed in triplicate.

#### 2.5.2. ATR-FTIR Spectroscopy and X-Ray Diffraction

ATR-FTIR spectra of chitosan, the extract, blank ChNPs and loaded ChNPs were recorded on an IRTracer-100 spectrometer with a QATR-10 single-reflection diamond module (Shimadzu Corporation, Kyoto, Japan) between 4000 and 650 cm^−1^ at 4 cm^−1^ resolution, accumulating 32 scans against a background collected under identical conditions. X-ray diffraction patterns were recorded on a MiniFlex 600 diffractometer (Rigaku, Tokyo, Japan) with Cu Kα radiation (λ = 1.5406 Å) at 40 kV and 15 mA, over a 2θ range of 5–60° at 1°/min with a step size of 0.02°. Spectra and diffractograms are representative of triplicate measurements.

#### 2.5.3. Determination of Encapsulation Efficiency

Encapsulation efficiency (%EE) was determined indirectly from the non-encapsulated phenolics recovered in the supernatant of the freshly prepared nanosuspension (Section 2.4), quantified by both the Folin–Ciocalteu assay (Section 2.3.1) and UHPLC-DAD (Section 2.3.3). No lyophilisation or washing step preceded this determination, so the measured non-encapsulated fraction reflects the freshly formed nanoparticles:

(1) %EE = [(*T* − *N*)/*T*] × 100

where *T* is the total mass of phenolics added to the formulation and *N* the mass recovered in the fresh supernatant. Any nanoparticles not sedimented under these conditions would increase *N* and lower the calculated %EE, so the reported values are conservative. Determinations were performed in triplicate.

#### 2.5.4. Thermogravimetric Analysis

Thermal decomposition behaviour of chitosan, blank ChNPs and loaded ChNPs were assessed on a SETSYS TG-DTA 16/18 system (Setaram Instrumentation, Lyon, France). Samples of 5.0 ± 0.5 mg were heated from 25 to 600 °C at 20 °C/min under an air flow of 50 mL/min. Analyses were performed in duplicate, and representative curves are presented.

#### 2.5.5. Morphological Characterisation

Nanoparticle morphology was examined by field-emission scanning electron microscopy (JSM-7610F, JEOL Ltd., Tokyo, Japan) at an accelerating voltage of 2 kV. Freeze-dried samples were mounted on aluminium stubs with double-sided carbon tape and sputter-coated with gold prior to imaging.

#### 2.5.6. In Vitro Antioxidant Activity of Nanoparticles

To release the encapsulated phenolics, freeze-dried AHE-loaded nanoparticles (10.0 mg) were dispersed in 5 mL of water containing 1% (*v*/*v*) acetic acid together with 5 mL of methanol and sonicated in an ice bath for 20 min; the acidic medium disrupted the crosslinked chitosan network, liberating the entrapped phenolic acids. The resulting solutions were analysed for total phenolic content (Section 2.3.1) and antioxidant activity by DPPH•, ABTS•^+^ and CUPRAC (Section 2.3.2). Blank ChNPs, a chitosan solution of equivalent concentration and the solvent mixture alone were processed identically and analysed as controls, and their responses were subtracted where appropriate. To permit direct comparison with the non-encapsulated extract, results are expressed per unit mass of the phenolics released from the powder (µmol TE/mg GAE). Analyses were performed in triplicate.

### 2.6. In Vitro Simulated Gastrointestinal Digestion

Simulated gastrointestinal digestion was performed following Peanparkdee et al. [26] with minor modifications through three consecutive phases (oral, gastric and intestinal). To deliver comparable phenolic loads, 50.0 mg of AHE-loaded ChNPs (1.5 mg GAE/mL formulation, corresponding to 2.27 mg GAE) and 3.0 mL of the free alkaline extract (corresponding to 2.03 mg GAE) were digested in parallel. The two loads differ because 50.0 mg of powder and 3.0 mL of extract were taken as the starting quantities, rather than amounts adjusted to a common phenolic content. Since recovery is referred to each sample’s own undigested content (Equation (2)), the results are expressed on a common relative basis despite the small difference in the initial absolute loads. All incubations were carried out at 37 °C in a shaking incubator at 120 rpm in the dark. Enzyme quantities are reported both as the mass of commercial preparation used and as the corresponding activity calculated from the supplier specifications in Section 2.1.2.

Oral phase. The sample was combined with 10 mL of simulated salivary fluid (SSF) and incubated for 10 min. SSF was prepared by dissolving 2.38 g of Na_2_HPO_4_, 0.19 g of KH_2_PO_4_, and 8.0 g of NaCl in 1 L of double-distilled water, adjusted to pH 6.75, and contained α-amylase (0.104 g; ≥52 U/mL). An aliquot was withdrawn and centrifuged (4696× *g*, 10 min, 4 °C), and the supernatant was stored at 4 °C until analysis.

Gastric phase. The remaining oral digesta was adjusted to pH 1.2 with 1 M HCl, combined with 10 mL of simulated gastric fluid containing pepsin (0.31% *w*/*v*; ≥250 U/mg) and incubated for 2 h. The digesta was then neutralised to pH 7.0 with 1 M NaOH, which inactivates pepsin. An aliquot was withdrawn and centrifuged (4696× *g*, 10 min, 4 °C), and the supernatant was stored at 4 °C until analysis.

Intestinal phase. To the neutralised digesta, 7.5 mL of simulated intestinal fluid (SIF), 1.25 mL of 120 mM NaCl, and 1.25 mL of 5 mM KCl were added, and the mixture was incubated for 2 h. SIF was prepared by dissolving 0.05 g of pancreatin (8× USP specification) and 0.3 g of porcine bile extract in 35 mL of 0.1 M NaHCO_3_, delivering approximately 10.7 mg of pancreatin and a final bile concentration of about 5 mM. Enzymatic activity was then arrested by placing the tubes on ice, and the mixture was centrifuged (4696× *g*, 10 min, 4 °C); the supernatant was stored at 4 °C until analysis. For the free-extract control, the identical procedure was followed, substituting the ChNPs with 3.0 mL of the undiluted alkaline extract at the oral phase.

Blank digestions containing all enzymes, bile and electrolytes but no sample were carried through the three phases in parallel and analysed by every assay; their responses were subtracted from the corresponding sample values to correct for the reactivity of the digestive proteins and bile salts in the Folin–Ciocalteu, ABTS and CUPRAC assays. The UHPLC-DAD determinations were not affected by this interference, as the enzymes did not co-elute with the target phenolic acids.

Supernatants from each phase were analysed for individual phenolic acids (UHPLC-DAD), total phenolic content (Folin–Ciocalteu) and antioxidant activity (ABTS, DPPH, and CUPRAC). Recovery (%R) after each phase was calculated as:

(2) %R = (C_e_/C_0_) × 100

where C_e_ is the blank-corrected concentration (or antioxidant capacity) in the supernatant after a given phase and C_0_ the corresponding value in the undigested sample. For the encapsulated samples, C_0_ was the total phenolic content released from the nanoparticles following their complete disruption by acidification, as described in Section 2.5.6. The supernatants from each phase were analysed in triplicate (*n* = 3), and results are reported as mean ± SD.

### 2.7. Statistical Analysis

All determinations were performed in triplicate, except for thermogravimetric analysis, which was carried out in duplicate; results are reported as mean ± standard deviation. Normality of the residuals was assessed by the Shapiro–Wilk test and homogeneity of variances by Levene’s test. Differences among nanoparticle formulations were evaluated by a one-way analysis of variance (ANOVA) followed by Tukey’s honestly significant difference test. Pairwise comparisons between encapsulated and free (non-encapsulated) samples within each digestion phase were performed using the Welch’s *t*-test. Differences were considered statistically significant at *p* < 0.05. All analyses were conducted using IBM SPSS Statistics v29 (IBM Corp., Armonk, NY, USA).

## 3. Results and Discussion

The three polar rice bran extracts were first characterised for their phenolic composition and antioxidant capacity to identify the most suitable one for encapsulation. The selected extract was then loaded into chitosan nanoparticles, which were characterised in terms of their physicochemical properties, encapsulation efficiency and antioxidant activity, and finally evaluated for the stability of the encapsulated phenolics under simulated gastrointestinal digestion.

### 3.1. Comparison Between Rice Bran Extracts

The total phenolic content (TPC) of the three polar extracts was determined using the Folin–Ciocalteu assay (Table 1).

**Table 1:** Phenolic composition and antioxidant activity of rice bran polar extracts.

Extract	TPC	ABTS^•+^	DPPH•	CUPRAC
	mg GAE/g RB	μmol TE/g RB	μmol TE/g RB	μmol TE/g RB
FE	3.27 ± 0.28 ^a^	15.94 ± 0.39 ^a^	6.63 ± 0.22 ^a^	18.55 ± 1.24 ^a^
AHE	4.58 ± 0.17 ^b^	10.28 ± 0.10 ^b^	7.42 ± 0.25 ^b^	37.98 ± 2.22 ^b^
ACHE	1.30 ± 0.07 ^c^	8.22 ± 0.51 ^c^	4.52 ± 0.15 ^c^	11.19 ± 0.64 ^c^

All data are mean ± SD of three analytical replicates (*n* = 3). FE: Free extract; AHE: alkaline hydrolysis extract; ACHE: acid hydrolysis extract; TPC: total phenolic content; GAE: gallic acid equivalent; TE: Trolox equivalent; RB: rice bran. Different letters within a column indicate significant differences (Tukey’s HSD, *p* < 0.05).

The three extracts differed significantly in phenolic content, with the alkaline-hydrolysed extract (AHE) showing the highest TPC (4.58 mg GAE/g RB), followed by the free extract (FE), whereas the acid-hydrolysed extract (ACHE) showed the lowest value. Quantitatively, AHE contained approximately 1.4-fold higher TPC than FE and 3.5-fold higher than ACHE, reflecting the superior efficiency of alkaline hydrolysis in releasing bound phenolics from the rice bran matrix. This is in line with previous reports showing that alkaline treatment effectively cleaves ester linkages between hydroxycinnamic acids and cell-wall polysaccharides [38].

**Figure 1:**
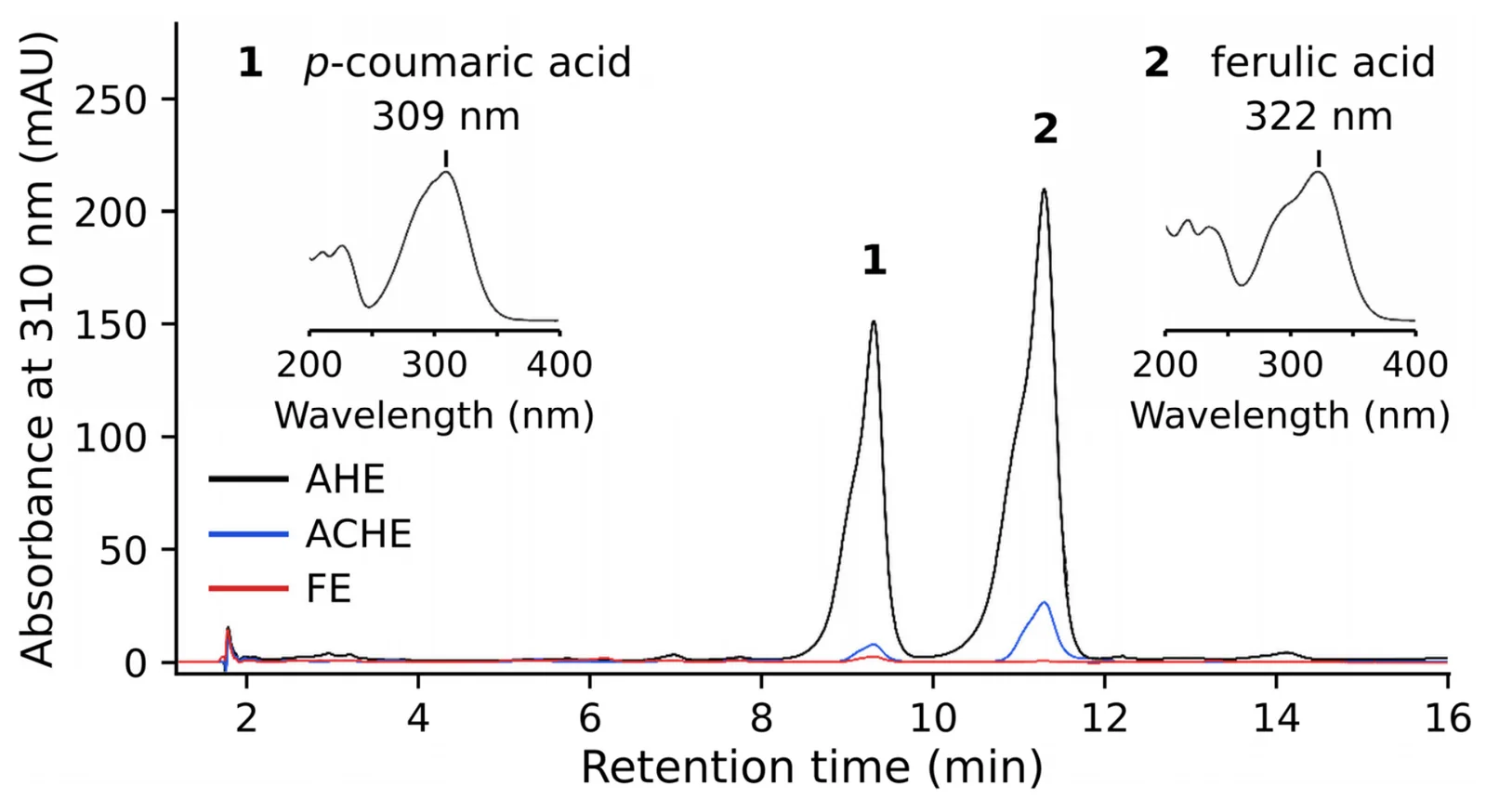
Chromatographic profile (310 nm) of rice bran polar extracts. FE: Free extract; AHE: alkaline hydrolysis extract; ACHE: acid hydrolysis extract.

UHPLC-DAD chromatographic analysis (Figure 1) further revealed pronounced compositional differences among the extracts. The hydrolysed extracts displayed similar qualitative profiles dominated by *p*-coumaric acid (t_R_ ≈ 9 min) and ferulic acid (t_R_ ≈ 11 min), in agreement with Irakli et al. [10].

AHE exhibited substantially higher peak intensities. Quantitative evaluation showed that AHE contained approximately 9-fold higher ferulic acid and 23-fold higher *p*-coumaric acid levels compared with ACHE, consistent with the greater effectiveness of alkaline hydrolysis for liberating ester-bound phenolic acids. This selectivity is consistent with the different lability of the two bond types: acid hydrolysis preferentially cleaves the ether bonds that anchor hydroxybenzoic acids to lignin, whereas alkaline hydrolysis targets the ester bonds through which hydroxycinnamic acids are attached to cell-wall polysaccharides [33,39]. In an earlier study on rice hulls, acid digestion under considerably harsher conditions (4 M HCl, 120 °C) gave extracts in which hydroxybenzoic acids and aldehydes, formed by degradation of the hydroxycinnamates, predominated [40]. Under the milder conditions applied here, the two acids remained the principal constituents of ACHE, albeit at low concentration, which points to incomplete cleavage of the ester bonds rather than to degradation of what had been released. In contrast, FE did not present detectable peaks corresponding to these two acids (below the limit of quantification of 2.5 and 2 ng for *p*-coumaric and ferulic acid, respectively), despite exhibiting measurable TPC and antioxidant activity. This apparent discrepancy reflects the well-recognised limitation of the Folin–Ciocalteu assay, which measures overall reducing capacity rather than phenolic specificity; the response of FE therefore probably arises from other endogenous reducing compounds.

The antioxidant activity assessed by ABTS•^+^, DPPH• and CUPRAC (Table 1) broadly followed the TPC trend, with AHE showing the highest activity, particularly in the CUPRAC assay. The discordance among the assays follows from the structures that dominate each extract rather than from the assays alone. AHE consists largely of *p*-coumaric and ferulic acid, and the behaviour of these two differs sharply from one assay to another. Toward DPPH• *p*-coumaric acid is essentially inactive and ferulic acid is only moderately active [35], which accounts for the modest DPPH• response of AHE relative to its phenolic content, whereas both acids scavenge ABTS•^+^ efficiently [41]. The same distinction governs the behaviour of the nanoparticles in Section 3.2.7. Notably, FE showed higher ABTS•^+^ activity than AHE (15.94 versus 10.28 µmol TE/g RB) despite its lower TPC and the absence of detectable ferulic and *p*-coumaric acid, which again points to non-phenolic reducing constituents in the non-hydrolysed extract. Although FE therefore exhibited measurable global antioxidant capacity, the markedly higher concentrations of the two principal hydroxycinnamic acids in AHE resulted in its selection as the most suitable candidate for the preparation of the chitosan nanoparticles described in the following sections.

### 3.2. Characterisation and Antioxidant Activity of the Chitosan Nanoparticles

#### 3.2.1. Particle Size, Distribution, Zeta Potential and Polydispersity Index

The conditions for nanoparticle formation by ionic gelation were established on the basis of the parameters known to determine the properties of chitosan–TPP systems, namely the concentrations of chitosan and sodium tripolyphosphate (TPP), their mass ratio, and the molecular weight and degree of deacetylation of the chitosan [14,42]. The chitosan used in the present study (viscosity-average molecular weight 190–310 kDa; degree of deacetylation >90%) is of medium molecular weight and high deacetylation, as reported for the same commercial product by Alexaki et al. [43]. Chitosan and TPP were used at 1.75 and 0.16 mg/mL, respectively, at a chitosan-to-TPP mass ratio of 3:1, in agreement with Pulicharla et al. [44]. This ratio has been reported as optimal for producing nanosized particles [37]: at a high chitosan-to-TPP ratio, TPP drives the crosslinking of the chitosan chains and favours the formation of smaller nanoparticles, whereas below this ratio the excess TPP promotes inter-particle bridging and aggregation. The high degree of deacetylation of the chitosan (>90%) provides many easily protonated amino groups available for crosslinking with TPP, consistent with the efficient particle formation obtained under these conditions [42].

The aim was not to obtain the smallest possible particles, but nanoscale particles with favourable polydispersity, zeta potential and encapsulation efficiency. Preliminary trials with the acid-hydrolysed extract (ACHE) did not yield particles within the nanoscale range, and this extract was in any case a poorer source of the two target acids than the alkaline hydrolysate (Section 3.1); only the alkaline extract (AHE) was therefore selected for encapsulation. The AHE-loaded nanoparticles were evaluated by DLS at three total phenolic levels (1.0, 1.5 and 2.0 mg GAE/mL), following Pulicharla et al. [44], who likewise encapsulated a phenolic plant extract by the same method (Table 2). The same table also reports the corresponding measurements for the nanoparticles prepared with the *p*-coumaric acid/ferulic acid standard mixture, formulated at the same concentration and ratio as the alkaline extract.

**Table 2:** Physicochemical properties of the chitosan nanoparticles at the three phenolic loadings, the blank and the standard mixture.

Formulation	Particle Size (nm)	PDI	Zeta Potential (mV)
AHE–1.0 mg GAE/mL	124.76 ± 6.88 ^a^	0.31 ± 0.03 ^a^	31.00 ± 1.08 ^a^
AHE–1.5 mg GAE/mL	184.69 ± 11.16 ^b^	0.35 ± 0.03 ^a,b^	32.77 ± 1.64 ^a^
AHE–2.0 mg GAE/mL	276.36 ± 15.32 ^c^	0.44 ± 0.02 ^b,c^	33.36 ± 1.58 ^a^
Blank	160.27 ± 14.32 ^b^	0.38 ± 0.02 ^b^	33.16 ± 1.69 ^a^
*p*-CA/FA–1.5 mg GAE/mL	126.43 ± 12.02 ^a^	0.34 ± 0.03 ^a,b^	33.85 ± 2.64 ^a^

All data are the mean ± SD of three analytical replicates (*n* = 3). AHE: Alkaline hydrolysis extract; *p*-CA/FA: *p*-coumaric acid/ferulic acid standards; PDI: polydispersity index. Different letters within a column indicate significant differences (Tukey’s HSD, *p* < 0.05).

Particle size increased with phenolic loading, from 124.76 nm at 1.0 mg GAE/mL to 184.69 nm at 1.5 mg GAE/mL and 276.36 nm at 2.0 mg GAE/mL, a trend consistent with Pulicharla et al. [44]. Notably, the particles obtained at the lowest loading (124.76 nm) were smaller than the blank (160.27 nm), so the size first decreased at low phenolic content before rising at higher loadings. At low concentration, the carboxyl groups of the hydroxycinnamic acids may act as additional crosslinking points that complement TPP and compact the polymer network, whereas at higher loadings the bulk incorporation of extract and partial neutralisation of the chitosan amino groups favour network expansion and larger particles [14].

The zeta potential, an indicator of surface charge and colloidal stability, remained high and positive for all formulations (+31.0 to +33.85 mV). Values beyond ±30 mV are generally associated with electrostatically stabilised dispersions, which is the relevant criterion here since no steric stabiliser was used [45]. Contrary to what might be expected, encapsulation did not reduce the zeta potential; the blank (+33.16 mV) and the AHE-loaded formulations (+31.0 to +33.36 mV) did not differ significantly (Table 2). The positive surface charge was retained on loading, which indicates that the phenolic acids did not accumulate at the particle surface in a form that neutralises it. At the working pH, their carboxyl groups are appreciably dissociated, and extensive surface association would therefore be expected to lower the positive potential. Zeta potential alone, however, cannot establish where the phenolics reside, and no claim of predominantly internal entrapment is made here. This high and stable charge is consistent with the high degree of deacetylation of the chitosan (>90%), which leaves a large fraction of amino groups protonated at the working pH [42] and agrees with values of approximately +33 mV reported for phenolic-loaded chitosan nanoparticles [46].

The polydispersity index (PDI) reflects the uniformity of the particle size distribution. Increasing the phenolic loading raised the PDI from 0.31 at 1.0 mg GAE/mL to 0.44 at 2.0 mg GAE/mL, with the blank at 0.38. According to Ding et al. [47], PDI values below 0.4 are considered acceptable; those authors likewise observed the highest polydispersity at the highest phenolic loading. In the present study, the criterion was met by the formulations loaded with up to 1.5 mg GAE/mL. A PDI of this order indicates a moderately narrow rather than a strictly monodisperse distribution, comparable with the value of 0.35 reported for an optimised chitosan nanoparticle formulation by Ghosh and Kuotsu [48]. The nanoparticles prepared with the standard mixture behaved similarly, although their mean size was approximately 1.5-fold smaller (126.43 vs. 184.69 nm), consistent with the absence of the co-extracted matrix constituents of the hydrolysate.

**Figure 2:**
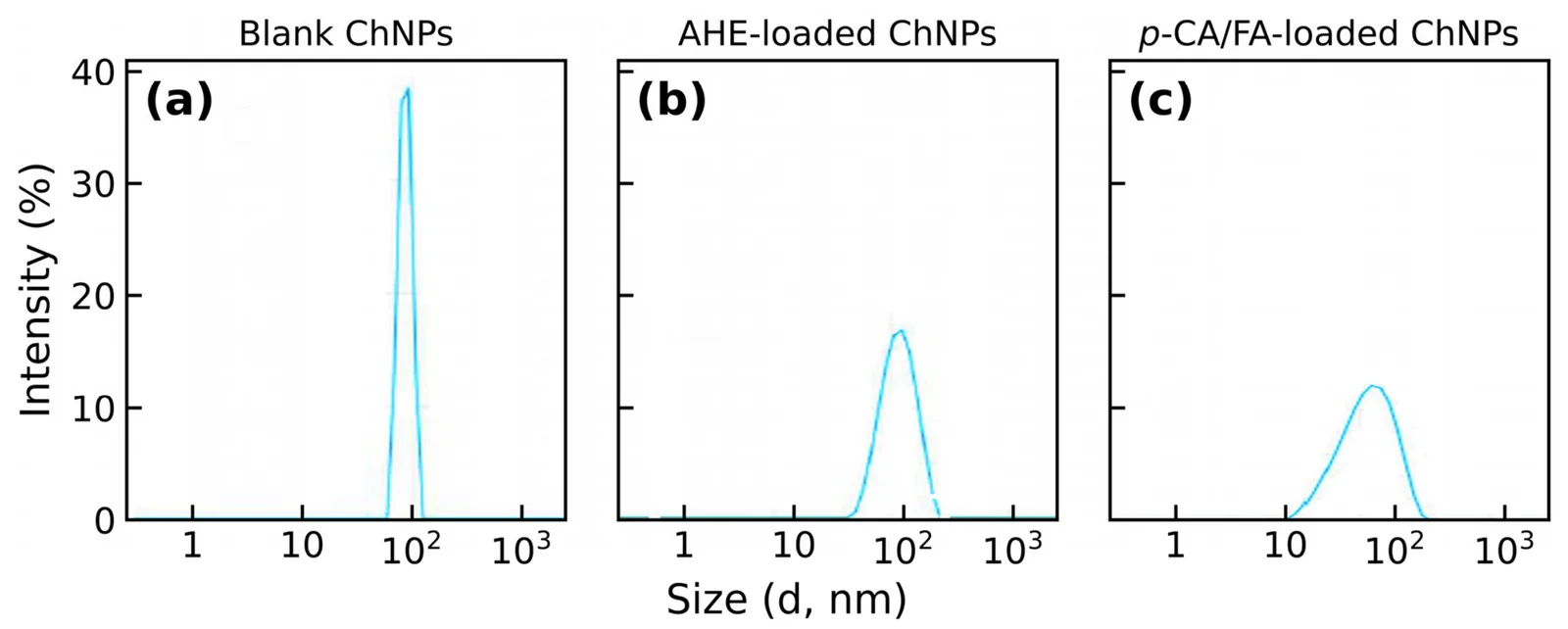
Intensity-weighted DLS size distributions of (**a**) blank chitosan nanoparticles, (**b**) nanoparticles loaded with the alkaline-hydrolysed extract at 1.5 mg GAE/mL and (**c**) nanoparticles loaded with the *p*-coumaric acid/ferulic acid standard mixture. The three panels share a common vertical scale.

The intensity-weighted size distributions (Figure 2) showed that, although the mean size broadened in the presence of either the extract or the mixture of the standard phenolic compounds, the distributions remained narrow and monomodal, with no secondary peaks, indicating a relatively uniform particle population that is favourable for the stability and reproducibility of the nanoparticles [42,49].

Based on these results, the polydispersity index was used as the primary criterion for selecting the phenolic loading used in the in vitro digestion study, the 1.5 mg GAE/mL formulation being the highest loading that satisfied the PDI < 0.4 criterion while remaining within the nanoscale range. As shown below, the same formulation also gave the highest encapsulation efficiency, so the two criteria converged on the same choice.

#### 3.2.2. Encapsulation Efficiency

The encapsulation efficiency of all formulations was determined indirectly, using both the Folin–Ciocalteu assay and UHPLC-DAD analysis (Table 3).

**Table 3:** Encapsulation efficiency (%EE) of the alkaline-hydrolysed extract at the three total phenolic loadings and of the *p*-coumaric/ferulic acid standard mixture.

Formulation	TPC (Colorimetric)	*p*-Coumaric Acid (Chromatographic)	Ferulic Acid (Chromatographic)
AHE—1.0 mg GAE/mL	27.83 ± 1.50 ^a^	33.48 ± 0.90 ^a^	25.24 ± 0.62 ^a^
AHE—1.5 mg GAE/mL	34.55 ± 1.92 ^b^	42.56 ± 3.65 ^b^	36.44 ± 3.51 ^b^
AHE—2.0 mg GAE/mL	14.87 ± 1.39 ^c^	34.11 ± 3.33 ^a^	19.84 ± 0.53 ^c^
*p*-CA/FA—1.5 mg GAE/mL	51.22 ± 3.80 ^d^	52.32 ± 3.94 ^c^	45.30 ± 4.10 ^d^

All data are the mean ± SD of three analytical replicates (*n* = 3). %EE: Encapsulation efficiency; AHE: alkaline hydrolysis extract; *p*-CA/FA: *p*-coumaric acid/ferulic acid standards; TPC: total phenolic content. Different letters within a column indicate significant differences (Tukey’s HSD, *p* < 0.05).

Both methods showed consistent trends, with the 1.5 mg GAE/mL formulation achieving the highest efficiency among the extract loadings (34.55% by the colorimetric method; 42.56% and 36.44% for *p*-coumaric and ferulic acid, respectively, by UHPLC-DAD). These values are in the range reported for natural phenolic extracts encapsulated in chitosan–TPP systems: Stoica et al. [37] obtained 25.8–46.0% for rose extract depending on the chitosan-to-TPP mass ratio, while Pulicharla et al. [44], using the same 3:1 ratio as the present study, reached a maximum of 58.48% for strawberry phenolics. The pronounced decline in efficiency at 2.0 mg GAE/mL, which coincides with the loss of colloidal quality at that loading (PDI 0.44), probably reflects saturation of the available crosslinking sites, beyond which additional extract remains in the continuous phase rather than being entrapped. Madureira et al. [50] described a competitive interaction between the hydroxyl groups of phenolic compounds and the phosphate groups of TPP for the protonated amino groups of chitosan, which reduces productive particle formation and is intensified at higher polyphenol contents. A comparable pattern of decreasing efficiency with increasing particle size at higher loading has also been reported for chlorogenic acid in chitosan nanoparticles [46]. On a dry-mass basis, the freeze-dried formulation prepared at 1.5 mg GAE/mL contained 45.4 ± 4.0 mg GAE per g of solid, that is 4.5% (*w*/*w*), determined after disruption of the particles by acidification (Section 2.5.6).

The synthetic mixture of *p*-coumaric and ferulic acid, formulated to match the composition of the extract, was encapsulated more efficiently than the natural extract itself (52.32% vs. 42.56% for *p*-coumaric acid; 45.30% vs. 36.44% for ferulic acid). Since the two formulations were identical except for the remaining constituents of the hydrolysate, the difference is attributed to the extract matrix. Those constituents were not identified individually, so the compounds responsible cannot be named. Their contribution is nonetheless substantial, since only about 3.6% of the alkaline extract was phenolic by Folin–Ciocalteu (Section 2.2.2), so that non-phenolic material accounted for the remainder of the mass introduced into the formulation. The partition into ethyl acetate at pH 2 restricts what that material can be, as sugars and peptides remain largely in the aqueous phase while acidic and lipophilic species are carried over. Free fatty acids released by saponification of the rice bran oil, which was not removed before hydrolysis, together with minor phenolic acids and other lipophilic constituents of the bran, are therefore the more likely candidates. This remains an inference; identifying the species responsible would require compositional characterisation of the hydrolysate together with encapsulation of the purified acids spiked with individual matrix components. To the best of our knowledge, the ionic gelation behaviour of a natural rice bran extract has not previously been compared directly with that of a matched synthetic mixture of its principal hydroxycinnamic acid constituents.

In every formulation, ferulic acid was entrapped less efficiently than *p*-coumaric acid. The encapsulation of phenolic acids in chitosan depends on their polarity and molecular characteristics, which determine the balance of associative and repulsive forces between the phenolic compounds and the chitosan chains; at the acidic working pH, the hydroxyl groups of the phenolic acids interact with the protonated amino groups of chitosan, and differences in polarity influence how readily each compound is retained within the polymer matrix [51]. Compound-dependent differences of this kind have been reported across a range of phenolics encapsulated in chitosan nanoparticles, with efficiencies of 48–59% for chlorogenic acid, 59–85% for gallic acid and 14.71–56.45% for ferulic acid [51]. The value obtained here for ferulic acid (36.44%) falls within that range, and its lower efficiency relative to *p*-coumaric acid is consistent with the additional methoxy substituent at C-3.

#### 3.2.3. Attenuated Total Reflectance Fourier-Transform Infrared Spectroscopy (ATR-FTIR)

**Figure 3:**
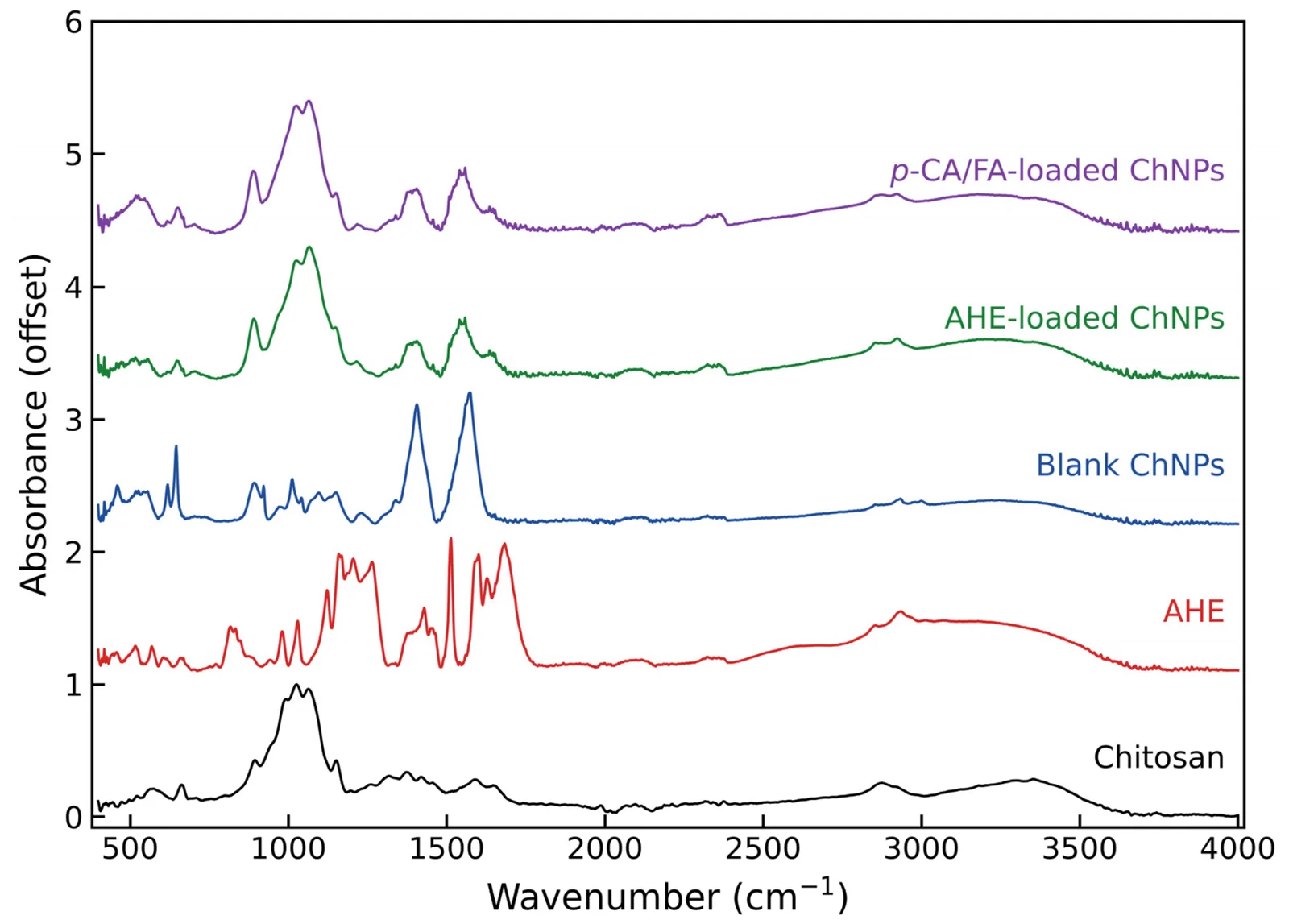
Comparative ATR-FTIR spectra of chitosan, the alkaline-hydrolysed rice bran extract (AHE) and the chitosan nanoparticles (blank and loaded forms). AHE: Alkaline hydrolysis extract; *p*-CA/FA: *p*-coumaric acid/ferulic acid standards.

The structural characterisation of chitosan, the selected rice bran extract and the corresponding nanoparticles was performed by ATR-FTIR spectroscopy, which provides information on the functional groups present and on the molecular interactions occurring during encapsulation. The attenuation, disappearance or shift of characteristic absorption bands is indicative of molecular entrapment and of interactions with the carrier, such as hydrogen bonding and electrostatic forces [52]. The spectrum of neat chitosan (Figure 3) exhibited a broad band at 3354 cm^−1^, attributed to overlapping O–H and N–H stretching and intermolecular hydrogen bonding, and a band at 2874 cm^−1^ corresponding to C–H stretching of the –CH_2_ and –CH_3_ groups [53]. The bands at 1647 cm^−1^, 1589 cm^−1^ and 1373 cm^−1^ were assigned to amide I, amide II (N–H bending) and the symmetric C–H deformation of the acetyl methyl group, respectively [49], while the bands at 1151 cm^−1^, 1063 cm^−1^ and 1026 cm^−1^ were associated with C–O and C–O–C stretching of the chitosan backbone [47,49].

Marked spectral changes were observed for the blank ChNPs (Figure 3). The broad band shifted from 3354 cm^−1^ to 3223 cm^−1^ and broadened, indicating modified hydrogen bonding on incorporation of TPP, in agreement with the shift from 3320 to 3268 cm^−1^ reported by da Silva et al. [52] for the same system. The amide I band shifted from 1647 cm^−1^ to 1573 cm^−1^, and a new band appeared at 1406 cm^−1^, consistent with the shifts reported on the crosslinking of chitosan with TPP and attributed to interaction between the amine groups of chitosan and the polyanionic phosphate groups of the crosslinker [54]; the band at 1573 cm^−1^ is assigned to amide II and that at 1406 cm^−1^ to the phosphate (P–O) groups of TPP [52]. The loss of the 1373 cm^−1^ band further reflects the rearrangement of chemical groups on chitosan–TPP interaction, in agreement with the disappearance of the corresponding bands reported by da Silva et al. [52]. An additional band at 893 cm^−1^ was attributed to P–O–P groups [53], consistent with electrostatic interaction between the NH_3_^+^ groups of chitosan and the phosphate groups of TPP; the intense absorption in the 1000–1150 cm^−1^ region reflects overlapping contributions from the C–O and C–O–C vibrations of the chitosan backbone and the P=O vibrations of TPP, which cannot be resolved individually.

The spectrum of the alkaline extract (AHE, Figure 3) showed characteristic bands at 3179 cm^−1^ and 2935 cm^−1^ (O–H and aromatic/aliphatic C–H stretching of ferulic and *p*-coumaric acid), a carbonyl (C=O) stretching band at 1683 cm^−1^ and aromatic ring (C=C) vibrations at 1600 cm^−1^ and 1513 cm^−1^. Further absorptions at 1429 cm^−1^ and 1264 cm^−1^ were attributed to =C–H bending [9] and to C–O stretching of the carboxylic groups of the phenolic acids [55], respectively.

The spectra of the AHE-loaded ChNPs (Figure 3) showed broader bands in the 3500–2500 cm^−1^ region relative to the blank, attributed to O–H···O bonding and indicating enhanced hydrogen bonding involving the phenolic –OH groups [37]. The C–H stretching band shifted from 2935 cm^−1^ to 2924 cm^−1^. The bands at 1573 cm^−1^ and 1406 cm^−1^ seen in the blank persisted at reduced intensity (1558 and 1406 cm^−1^, respectively; [56]), while intense bands at 1067 cm^−1^ and 1022 cm^−1^ were consistent with the incorporation of the extract, the changes in band intensity and position relative to the free extract suggesting entrapment within the chitosan–TPP matrix.

The nanoparticles loaded with the *p*-coumaric/ferulic acid standard mixture (Figure 3) gave spectra closely like those of the AHE-loaded nanoparticles. The comparable peak positions and shifts indicate that the phenolic acids and the natural extract interact with the chitosan–TPP network through analogous mechanisms, giving rise to similar structural modifications and consistent with encapsulation in both cases.

#### 3.2.4. Thermogravimetric Analysis (TGA)

**Figure 4:**
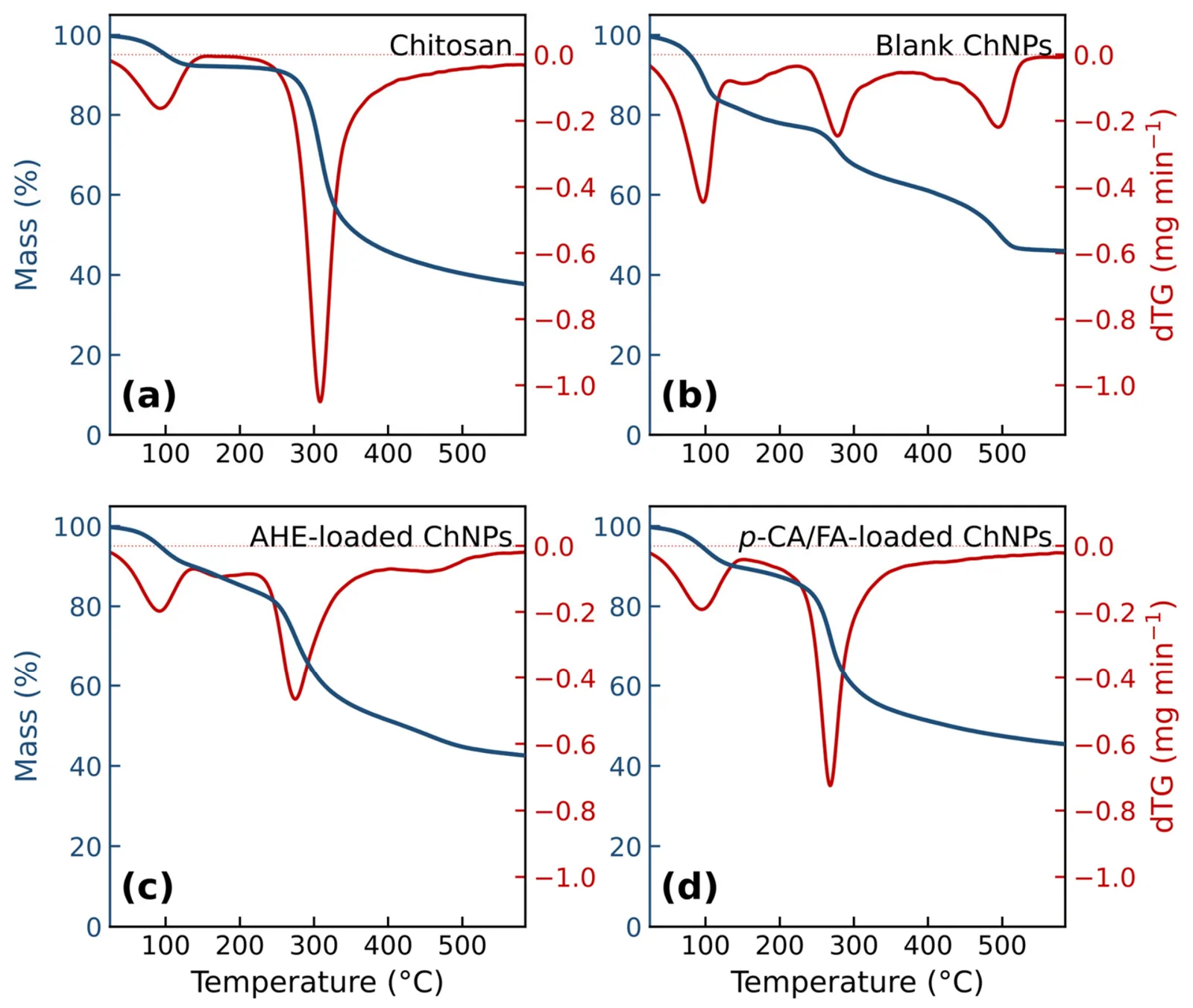
Thermogravimetric (TG, blue) and derivative thermogravimetric (dTG, red) curves of (**a**) chitosan, (**b**) blank ChNPs, (**c**) AHE-loaded ChNPs and (**d**) ChNPs loaded with the *p*-coumaric/ferulic acid standard mixture. AHE: Alkaline hydrolysis extract.

Thermogravimetric analysis was used to compare the decomposition behaviour of chitosan and of the prepared nanoparticles. Representative thermogravimetric (TG) and derivative thermogravimetric (DTG) curves are presented in Figure 4, in which the TG curve records the percentage mass loss with increasing temperature and the DTG curve, its first derivative, identifies the temperatures of maximum decomposition rate.

Neat chitosan exhibited two main degradation stages (Figure 4a). The first, between 50 and 140 °C, corresponded to the loss of unbound moisture (7.8%), while the second, between 260 and 400 °C, was attributed to degradation of the polymer backbone, with a maximum decomposition rate at 308 °C and a residual mass of 37.5%.

The blank chitosan nanoparticles (Figure 4b) showed a considerably greater initial moisture loss (18.9%), consistent with the additional water associated with the hydrogen bonding introduced upon ionic gelation. In contrast to neat chitosan, three degradation stages were observed, and the residual mass increased to 45.9%. The AHE-loaded ChNPs (Figure 4c) lost markedly less moisture (10.8%), consistent with the phenolic acids occupying hydrogen bonding sites that would otherwise retain water; their main degradation step occurred at 272 °C, and the residual mass was 43.9% (41.2% for the second replicate). The particles loaded with the *p*-CA/FA standard mixture (Figure 4d) behaved similarly with respect to moisture (10.4%) and to the position of the main degradation step (268 °C) but retained a higher residual mass (45.3%) and showed a sharper decomposition maximum, consistent with the more uniform composition of the synthetic mixture.

All three nanoparticle formulations showed altered thermal decomposition behaviour relative to neat chitosan, characterised by a higher residual mass (41–46% versus 37.5%) and by a much lower rate of backbone degradation (−0.25 to −0.73 versus −1.05 mg/min). The higher residual mass is in line with the formation of a more crosslinked network, attributed mainly to the phosphate groups introduced by TPP [49]. At the same time, the temperature of maximum decomposition shifted downward relative to neat chitosan (from 308 to 268–278 °C); this is due to the lower effective acetylation of the chitosan following its interaction with TPP [56] and indicates that crosslinking also altered the temperature dependence of the degradation process. A comparable downward shift in the first degradation step has likewise been reported alongside an overall gain in thermal stability [49].

The residual masses obtained here fall within the range reported for comparable systems. Alehosseini et al. [49] recorded total weight losses of 63.6% for chitosan and 52.0% for blank chitosan nanoparticles, corresponding to residual masses of approximately 36% and 48% and to the same direction of change observed here. Da Silva et al. [52] reported 31% for blank chitosan–TPP nanoparticles and 22.6% for the same particles loaded with a phenolic-rich by-product extract. A further difference between the blank and the loaded nanoparticles in the present study was the high-temperature degradation event observed in the blank at approximately 494 °C. Such an event was largely absent from both loaded formulations, suggesting that the presence of the phenolic acids alters the late-stage decomposition of the chitosan–TPP network.

#### 3.2.5. X-Ray Diffraction (XRD) Analysis

**Figure 5:**
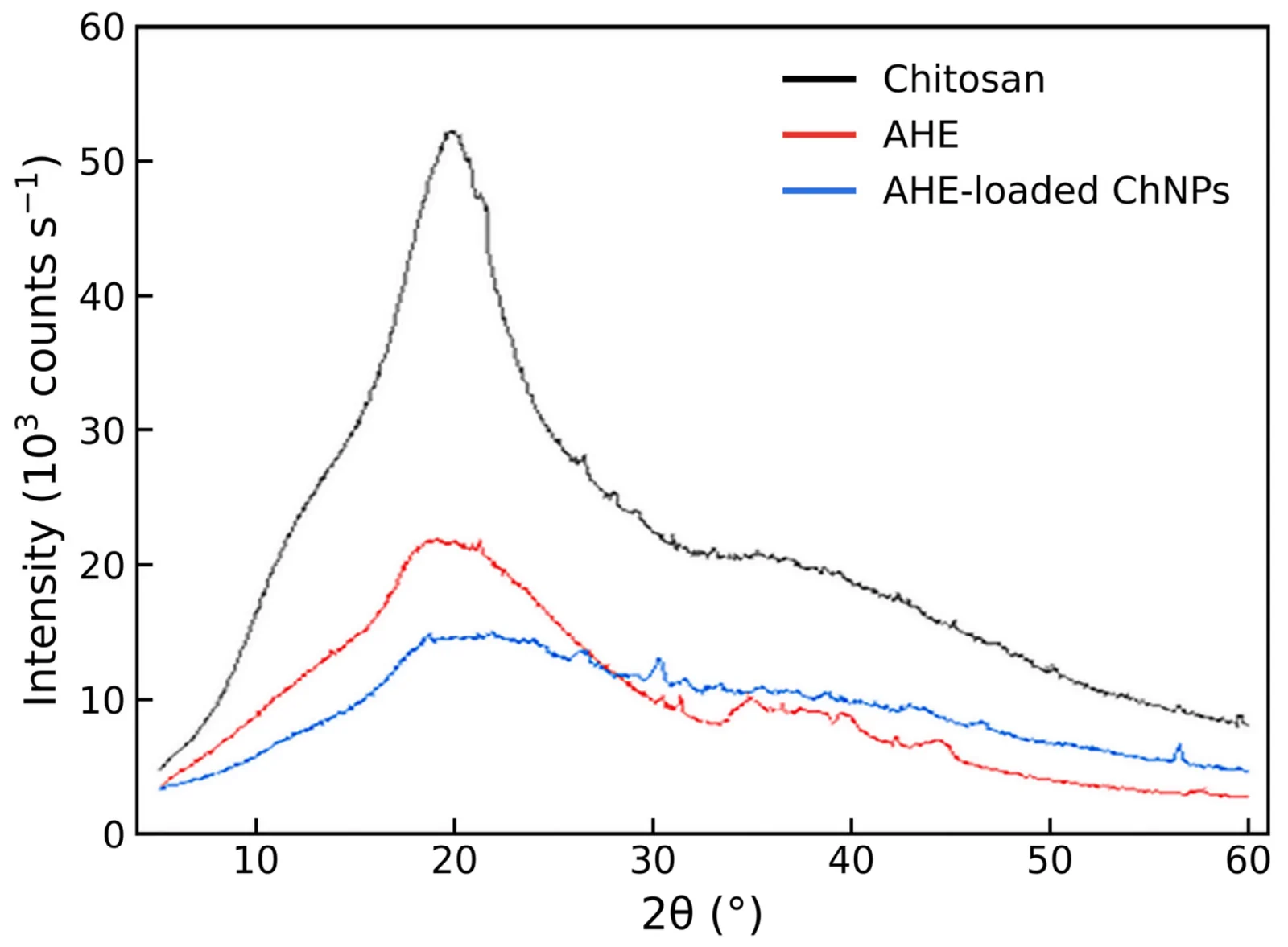
X-ray diffractograms of chitosan, the alkaline-hydrolysed rice bran extract (AHE) and the AHE-loaded chitosan nanoparticles. AHE: Alkaline hydrolysis extract.

X-ray diffraction was used to assess changes in crystalline order on nanoparticle formation and encapsulation. Chitosan is semicrystalline, with reflections commonly reported at 2θ ≈ 11° and 21°; here, it gave a broad reflection centred at 2θ ≈ 20° (Figure 5).

The rice bran extract was predominantly amorphous, with a broad reflection at 2θ ≈ 19° and several weak features across the diffractogram. The extract-loaded nanoparticles retained only a broad, diffuse reflection near 2θ ≈ 20°, broader and less well defined than that of neat chitosan; the weak sharp features at higher angles are attributed to residual crystalline material. The lower absolute intensity of the loaded sample follows the same trend but is not taken as evidence, since intensity depends on sample quantity and packing.

The reduction in crystalline order is attributed to ionic crosslinking with TPP, which restricts chain mobility and hinders ordered packing. Similarly, Soleymanfallah et al. [57] found the amorphous character of blank chitosan nanoparticles to be retained after encapsulation. Ding et al. [47] reported the absence of distinct crystalline reflections in ionically gelled chitosan nanoparticles, which they interpreted as intimate incorporation of the loaded compound. The extensive hydrogen bonding indicated by ATR-FTIR (Section 3.2.3) agrees with this.

#### 3.2.6. Morphological Characterisation of the ChNPs

**Figure 6:**
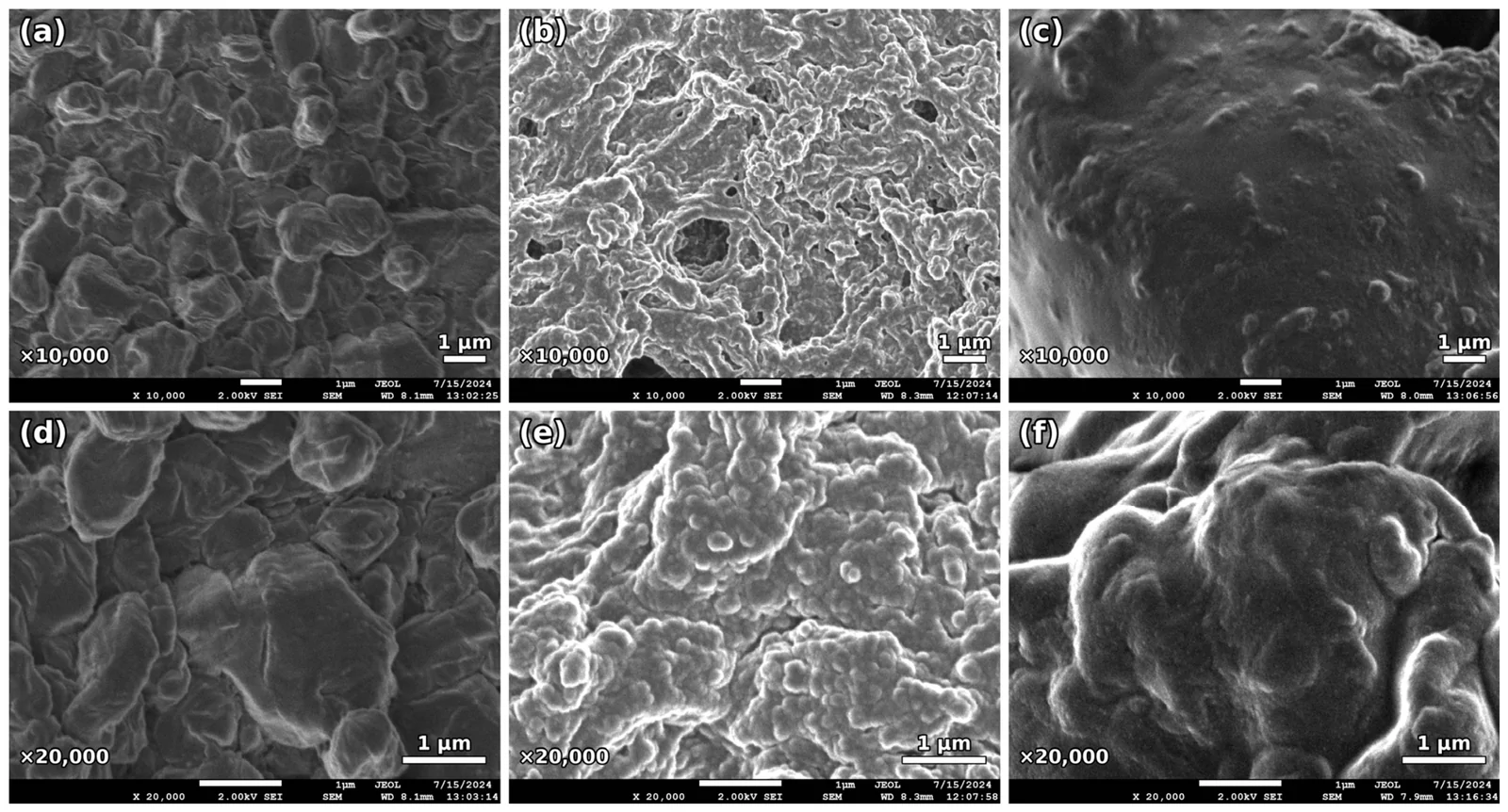
Scanning electron micrographs of the chitosan nanoparticles at matched magnifications: (**a**–**c**) ×10,000 and (**d**–**f**) ×20,000. (**a**,**d**) Blank nanoparticles; (**b**,**e**) AHE-loaded nanoparticles; (**c**,**f**) nanoparticles loaded with the *p*-coumaric/ferulic acid standard mixture. Scale bars: 1 µm. AHE: Alkaline hydrolysis extract.

The morphology of the blank and loaded nanoparticles was examined by scanning electron microscopy, with all three formulations recorded at the same two magnifications (Figure 6).

The blank ChNPs (Figure 6a,d) appeared as flattened, plate-like domains of one to two micrometres with smooth surfaces and sharp edges, in which individual particles could not be resolved. Freeze-drying was carried out without a cryoprotectant, and such coalescence is favoured by hydrogen bonding between adjacent particles and by ionic bridging through surface-bound TPP [49].

The AHE-loaded ChNPs (Figure 6b,e) instead retained a discrete particulate morphology, as densely packed, approximately spherical units of 100–200 nm. This matches the hydrodynamic diameter from DLS (184.7 nm; Section 3.2.1), which is not always the case, since SEM measures the dried state and usually gives smaller dimensions [49,54].

The particles loaded with the *p*-CA/FA mixture (Figure 6c,f) were also particulate but more extensively fused, with a finely granular rather than clearly discrete surface. Both loaded formulations therefore preserved nanoscale structure where the blank carrier did not, which may relate to the additional crosslinking contributed by the carboxyl groups of the hydroxycinnamic acids ([14]; Section 3.2.1).

#### 3.2.7. Antioxidant Activity of the Nanoparticles

The antioxidant activity of the encapsulated phenolics was assessed after their release from the nanoparticles and compared with that of the free extract. The phenolic acids account for only a fraction of the particle mass. Each response was therefore normalised to the measured phenolic content of the sample and expressed as specific activity, so that encapsulation efficiency does not enter the comparison.

CUPRAC gave a comparable reducing capacity for the encapsulated material and the free extract: approximately 11 versus 10 µmol TE mg^−1^ GAE. The Folin–Ciocalteu assay served as the normalising basis, not as an independent measure of activity. Neither the blank nanoparticles nor the chitosan solution responded in any assay. Ding et al. [47] report the same for blank chitosan–TPP nanoparticles, where the crosslinker masks the amino groups that would otherwise react. Normalisation was based on the phenolic content of the freeze-dried solid, 45.4 ± 4.0 mg GAE/g. Encapsulation therefore preserved the reducing capacity of the two acids, as Nallamuthu et al. [46] also found for chlorogenic acid in the same carrier.

Neither radical-based assay was informative for the undigested nanoparticles, and for different reasons. DPPH• failed because *p*-coumaric acid is essentially inactive toward this radical and ferulic acid is only moderately active [35]. ABTS•^+^ cannot be explained this way; both acids scavenge ABTS•^+^ efficiently, and *p*-coumaric acid is as active as rosmarinic acid on a Trolox-equivalent basis [41]. The more likely reason is that the entrapped acids were not accessible to the radical cation; and indeed, ABTS•^+^ became informative once the particles were disrupted during digestion (Section 3.3). Soltanzadeh et al. [54] and Madureira et al. [58] reached the same conclusion for chitosan nanoparticles loaded with pomegranate peel extract and with phenolic acids, respectively.

No single assay settles antioxidant activity; assays differ in reaction mechanism, target species and conditions [59]. The reducing assays were therefore taken as the primary descriptors. Section 3.3 examines what happens to the encapsulated phenolics under simulated gastrointestinal conditions.

### 3.3. Behaviour During Simulated Gastrointestinal Digestion

At each phase of the simulated digestion, the recovery (%R) of the individual acids (*p*-coumaric and ferulic acid, by UHPLC-DAD) of total phenolic content (Folin–Ciocalteu) and of antioxidant activity (ABTS•^+^ and CUPRAC) was determined for the AHE-loaded nanoparticles and for the free extract (Table 4). DPPH• gave no measurable signal in the digesta, as expected from the very low intrinsic DPPH• activity of the two acids (Section 3.2.7), and was excluded from the series; the same was observed in a second, independent digestion run. Blank nanoparticles and the plain chitosan solution gave no detectable response in any assay. ABTS•^+^ was retained because recovery is referred to each sample’s own initial content, so the phase-to-phase ratio holds even where the absolute signal is modest.

**Table 4:** Recovery (%R) of the AHE-loaded chitosan nanoparticles and the free extract during the three phases of simulated gastrointestinal digestion (oral, gastric and intestinal).

Phase	Sample	*p*CA	FA	TPC	CUPRAC	ABTS
Oral	AHE-loaded ChNPs	95.4 ± 4.5 ^a^	93.1 ± 3.5 ^a^	71.9 ± 3.5 ^a^	67.5 ± 6.1 ^a^	57.3 ± 4.1 ^a^
	Free extract	84.2 ± 0.6 ^b^	76.7 ± 0.6 ^b^	45.0 ± 1.2 ^b^	13.0 ± 0.1 ^b^	23.5 ± 0.5 ^b^
	*p*-value	0.048	0.013	0.0027	0.0041	0.0044
Gastric	AHE-loaded ChNPs	53.6 ± 4.5 ^a^	49.0 ± 3.0 ^a^	50.7 ± 1.0 ^a^	35.7 ± 1.8 ^a^	24.2 ± 1.5 ^a^
	Free extract	42.2 ± 0.1 ^b^	38.3 ± 0.1 ^b^	26.5 ± 1.5 ^b^	6.4 ± 0.1 ^b^	11.2 ± 0.5 ^b^
	*p*-value	0.048	0.025	6.0 × 10^−5^	0.0012	0.0021
Intestinal	AHE-loaded ChNPs	24.2 ± 1.0 ^a^	22.1 ± 0.8 ^a^	43.1 ± 1.0 ^a^	21.5 ± 0.7 ^a^	12.2 ± 0.4 ^a^
	Free extract	5.8 ± 0.01 ^b^	5.4 ± 0.01 ^b^	20.8 ± 0.8 ^b^	4.3 ± 0.4 ^b^	8.0 ± 0.5 ^b^
	*p*-value	9.8 × 10^−4^	7.6 × 10^−4^	1.1 × 10^−5^	2.7 × 10^−5^	4.4 × 10^−4^

Values are the mean ± SD of three analytical replicates (*n* = 3). Within each phase, different superscript letters (a,b) indicate a statistically significant difference between the encapsulated and the free form; exact *p*-values from Welch’s *t*-test are given beneath each pair. pCA: *p*-coumaric acid; FA: ferulic acid; TPC: total phenolic content (Folin–Ciocalteu); CUPRAC and ABTS: antioxidant capacity assays. Recovery (%R) is expressed relative to each sample’s own undigested content.

In the oral phase, the encapsulated extract recovered more than the free extract for every parameter, all differences significant (Welch’s *t*-test, *p* < 0.05).

In the gastric phase, the same trend held. The largest relative difference was in CUPRAC (5.6-fold), the strongest statistical separation in total phenolic content (*p* = 6.0 × 10^−5^). Peanparkdee et al. [26], whose protocol was followed here, reported gastric release below 20% for the free extracts of two non-coloured Thai cultivars against markedly higher values for the same extracts in gelatine.

The intestinal phase gave the clearest result. Recovery of *p*-coumaric and ferulic acid from the nanoparticles was roughly 4-fold that from the free extract (24.2% vs. 5.8% and 22.1% vs. 5.4%), and CUPRAC followed the same pattern (21.5% vs. 4.3%). The ABTS•^+^ advantage was smaller (12.2% vs. 8.0%), in keeping with the low absolute response of this system. All differences in this phase were significant at *p* < 0.001. The phenolic acid recoveries come from chromatographic quantification of the individual compounds, and the colorimetric and antioxidant data agree with them.

Absolute recoveries of total phenolic content were lower than the 70–85% reported by Peanparkdee et al. [26] for gelatine-encapsulated rice bran extract, whose release is referred to the extract added to the preparation rather than to the content of the particles. Peanparkdee et al. [60] used the same basis as in the present study and noticed a decline from the gastric to the intestinal phase, which they attributed to the instability of phenolics near neutral pH. Two chitosan studies point the same way. In one, García-Carrasco et al. [61] found higher reducing and Trolox-equivalent capacity after intestinal digestion for encapsulated than for free oregano polyphenols. In the second, Madureira et al. [58] found most of the phenolic load released in the intestinal rather than the gastric phase. The present data establish that a larger fraction of the hydroxycinnamic acids is recovered in the intestinal digesta from the nanoparticles than from the free extract. Because the recovery measured after each phase combines release from the carrier with degradation in the digestive medium, it does not on its own demonstrate improved chemical stability or controlled release. Bioaccessibility is the fraction released and made available for absorption, not the fraction absorbed; systemic bioavailability would require a permeability assay or an in vivo model [62].

Release kinetics were therefore not modelled; separating the two contributions would require a time-resolved design under non-degradative conditions. Long-term colloidal stability was not evaluated either, nor was the stability of the free and the encapsulated acids followed under storage, which would be the direct test of a protective effect.

## 4. Conclusions

An alkaline-hydrolysed rice bran extract enriched in ferulic and *p*-coumaric acids was encapsulated in chitosan nanoparticles by ionic gelation with tripolyphosphate. Alkaline hydrolysis released far more of the two acids than aqueous ethanolic or acidic extraction. The particles prepared at the highest loading that remained nanoscale, 1.5 mg GAE/mL, were monomodal and highly positively charged, and the charge was retained on loading. Electron microscopy showed discrete particles of a size matching the light-scattering data, while the blank carrier coalesced on drying. FTIR, XRD and TGA indicated entrapment, reduced crystallinity and altered thermal decomposition relative to chitosan.

A matched synthetic mixture of the two acids was encapsulated more efficiently than the extract, which points to an inhibitory effect of the extract matrix, although the constituents responsible were not identified. Per unit of phenolic content, the reducing capacity of the formulation matched that of the free extract, so encapsulation did not diminish the intrinsic activity of the acids. Under simulated digestion, their intestinal recovery was about 4-fold that of the free extract. Whether that higher recovery reflects protection against degradation, modified release, or both was not resolved here. The work is limited to in vitro bioaccessibility; intestinal absorption would require a permeability assay or an in vivo model. Within that limit, chitosan nanoparticles are a promising vehicle for valorising rice bran hydroxycinnamic acids.

## Data Availability

.The original contributions presented in this study are included in the article. Further inquiries can be directed to the corresponding author.
